# Alternating Red and Blue Light-Emitting Diodes Allows for Injury-Free Tomato Production With Continuous Lighting

**DOI:** 10.3389/fpls.2019.01114

**Published:** 2019-09-13

**Authors:** Jason Lanoue, Jingming Zheng, Celeste Little, Alyssa Thibodeau, Bernard Grodzinski, Xiuming Hao

**Affiliations:** ^1^Department of Plant Agriculture, University of Guelph, Guelph, ON, Canada; ^2^Harrow Research and Development Centre, Agriculture & Agri-Food Canada, Harrow, ON, Canada

**Keywords:** continuous lighting, light-emitting diodes, spectral quality, tomato, photoperiod, net carbon exchange rate, greenhouse, supplemental lighting

## Abstract

Plant biomass is largely dictated by the total amount of light intercepted by the plant [daily light integral (DLI) — intensity × photoperiod]. Continuous light (CL, 24 h lighting) has been hypothesized to increase plant biomass and yield if CL does not cause any injury. However, lighting longer than 18 h causes leaf injury in tomato characterized by interveinal chlorosis and yield is no longer increased with further photoperiod extension in tomatoes. Our previous research indicated the response of cucumbers to long photoperiod of lighting varies with light spectrum. Therefore, we set out to examine greenhouse tomato production under supplemental CL using an alternating red (200 µmol m^−2^ s^−1^, 06:00–18:00) and blue (50 µmol m^−2^ s^−1^, 18:00–06:00) spectrum in comparison to a 12 h supplemental lighting treatment with a red/blue mixture (200 µmol m^−2^ s^−1^ red + 50 µmol m^−2^ s^−1^ blue, 06:00–18:00) at the same DLI. Our results indicate that tomato plants grown under supplemental CL using the red and blue alternating spectrum were injury-free. Furthermore, parameters related to photosynthetic performance (i.e., Pn_max_, quantum yield, and F_v_/F_m_) were similar between CL and 12 h lighting treatments indicating no detrimental effect of growth under CL. Leaves under CL produced higher net carbon exchange rates (NCER) during the subjective night period (18:00–06:00) compared to plants grown under 12 h lighting. Notably, 53 days into the treatment, leaves grown under CL produced positive NCER values (photosynthesis) during the subjective night period, a period typically associated with respiration. At 53 days into the growth cycle, it is estimated that leaves under CL will accumulate approximately 800 mg C m^−2^ more than leaves under 12 h lighting over a 24 h period. Leaves grown under CL also displayed similar diurnal patterns in carbohydrates (glucose, fructose, sucrose, and starch) as leaves under 12 h lighting indicating no adverse effects on carbohydrate metabolism under CL. Taken together, this study provides evidence that red and blue spectral alternations during CL allow for injury-free tomato production. We suggest that an alternating spectrum during CL may alleviate the injury typically associated with CL production in tomato.

## Introduction

Continuous light (CL) means a constant flux of energy into photosynthesis, theoretically leading to increased growth and the potential for higher yield. In lettuce, low irradiance CL has already been shown to increase plant growth (Kitaya et al., 1998; Ohtake et al., 2018). However, in plants such as tomato, potato, and eggplant, CL has been associated with negative responses in growth traits (Velez-Ramirez et al., 2011). In tomatoes, a down regulation in photosynthesis due to excess accumulation in carbohydrates, decreased maximum quantum efficiency of photosystem II (PSII), and early leaf senescence has been reported during extended photoperiods leading to CL-injury such as chlorosis and decreased production (Demers et al., 1998; Velez-Ramirez et al., 2017a).

The underlying mechanism of CL-injury in tomato has yet to be determined. However, comparisons between CL-tolerant wild-type tomatoes and CL-sensitive domesticated tomatoes *via* RNA sequencing has shown that a downregulation of the gene *type III light harvesting chlorophyll a/b binding protein 13* (*cab13*) during CL confers injury (Velez-Ramirez et al., 2014). Furthermore, differences (mis-matching) between the external light/dark cycles and a plant’s internal circadian rhythms has been demonstrated to decrease the photosynthetic rate and show injuries related to CL (Velez-Ramirez et al., 2017b). The role of circadian asynchrony in CL-injury is strengthened by the circadian oscillations of light harvesting complex genes as determined by mRNA analysis (Kellmann et al., 1993). Such oscillations may not allow the plant to fully utilize the CL during periods of low gene expression of proteins which make up the light harvesting complex.

The role of photoreceptors (i.e., phytochrome and cryptochrome) in CL-injury has also been postulated (Velez-Ramirez et al., 2011). Demers and Gosselin (2000) noted that tomato grown under CL with metal halide (MH) lamps had more severe leaf chlorosis and decreased photosynthetic rates compared to plants grown under CL with high pressure sodium (HPS). Of note, MH luminaries have a higher blue light component than HPS indicating that spectral quality may play a role in CL-injury. A recent study indicates that the overexpression of phytochrome A diminishes the injury associated with CL in tomato (Velez-Ramirez et al., 2019). This result indicates a potential for spectral modifications in alleviating CL-injury.

In addition, the role of temperature has been investigated with respect to CL-injury. The presence of a thermoperiod (oscillations in temperature during a 24 h period) has been shown to negate the effect of CL-injury in many species (Hillman, 1956; Velez-Ramirez et al., 2011; Matsuda et al., 2014; Haque et al., 2017; Hao et al., 2017a; Hao et al., 2018b). In tomato, a temperature drop of 10°C during what would be the night period during CL increased maximum quantum efficiency of photosystem II (F_v_/F_m_) to levels similar to a control tomato plant grown under a conventional 12 h day/12 h night period (Haque et al., 2017). Using F_v_/F_m_ as an indication of photoinhibition (i.e., degradation of photosystem II antenna complex), an increase in this value indicates a reduction in CL-injury facilitated by a temperature drop. In addition, a temperature drop has been shown to alleviate the inhibition on photosynthesis due to excess carbohydrate production during CL (Demers and Gosselin, 1999; Matsuda et al., 2014; Haque et al., 2015; Haque et al., 2017). The carbohydrate status (glucose, fructose, sucrose, and starch) at the end of the light period from tomatoes grown under CL and a variable temperature (CLVT) was similar to plants grown under CL and a constant temperature (CLCT; Haque et al., 2017). Interestingly, at the end of what would have been the night period under CLVT, there was an increase in starch, sucrose, glucose, and fructose compared to the end of the day values (Haque et al., 2017). These results coupled with a high photosynthetic rate from plants under CLVT indicates that the accumulation of carbohydrates may not be solely responsible for CL-injury as it was not observed in CLVT treatment. Instead, it has been hypothesized that a temperature drop during CL may upregulate *cab13* allowing for normal energy balance and CL-injury free growth (Haque et al., 2017).

Most studies to date have used sole artificial CL with constant temperature and spectrum in controlled growth chambers and observed CL related injury in tomato (Hillman, 1956; Globig et al., 1997; Matsuda et al., 2014; Haque et al., 2015; Haque et al., 2017; Velez-Ramirez et al., 2017b). The exceptions (Arthur et al., 1930; Demers et al., 1998) used high intensity discharge lights with fixed light spectral composition as supplemental lighting within a greenhouse. However, due to the intrinsic properties of the lighting fixtures, temperature control was an issue and thus thermoperiod effects are likely to play a factor in the results obtained. Also, our previous research on cucumbers shows that response to long photoperiods including CL varies with light spectrum (Hao et al., 2018c).

The introduction of light-emitting diodes (LEDs) as a supplemental lighting fixture not only allows for better temperature control within greenhouses, but also provides the ability to administer time-dependent wavelength specific light during the photoperiod. Furthermore, using LEDs allows for the economical implementation of lighting fixtures which can alter spectral quality without the need for added units. Light capture is crucial in early stage of greenhouse vegetable growth when plant canopy is small (Hao et al., 2018a). Pure blue light (100%) can increase plant height and light capture in young cucumber plants although increasing proportion of blue light reduces plant height when red light or other spectrum of light is present (Hernández and Kubota, 2016). Red LEDs have high efficiency in generating photosynthetically active radiation (https://www.canr.msu.edu/uploads/resources/pdfs/red-light.pdf). Therefore, we set out to assess the physiological and morphological effects and fruit yield of supplemental CL with alternating red and blue (100% during the night) spectrum provided by LED fixtures.

The objective of the study was to assess the effects of supplemental CL with alternating red and blue light spectrum on tomato growth. We hypothesize that by alternating the light spectrum and providing 100% blue light during the night, CL-injury may be alleviated and light capture may be increased in tomato production. The hypothesis was tested during the winter months in a Canadian greenhouse when supplemental lighting is most needed to achieve adequate tomato production.

## Materials and Methods

### Plant Material and Experimental Design

Tomato (*Solanum lycopersicum*) seedlings cv. ‘Endeavour’ were grafted onto cv. ‘Maxifort’ in a double stemmed system. Transplants (5 weeks old—seeded on Oct. 2, 2018 and grafted on October 14, 2018) were placed into two adjacent double layers polyethylene (one year old) greenhouses (50 m^2^ growing area) at the Harrow Research and Development Centre, Agriculture and Agri-Food Canada, Harrow, Ontario, Canada (42.03°N, 82.9°W) on November 9, 2018 at a plant density of 1.75 plants m^−2^ (3.5 stems m^−2^). The plants were drip-irrigated using a complete nutrient solution (Ontario Ministry of Agriculture, Food and Rural Affairs (Ontario Ministry of Agriculture, Food and Rural Affairs, 2010). The EC and pH were set at 2.8 dS m^−1^ and 5.8 respectively.

**Figure 1:**
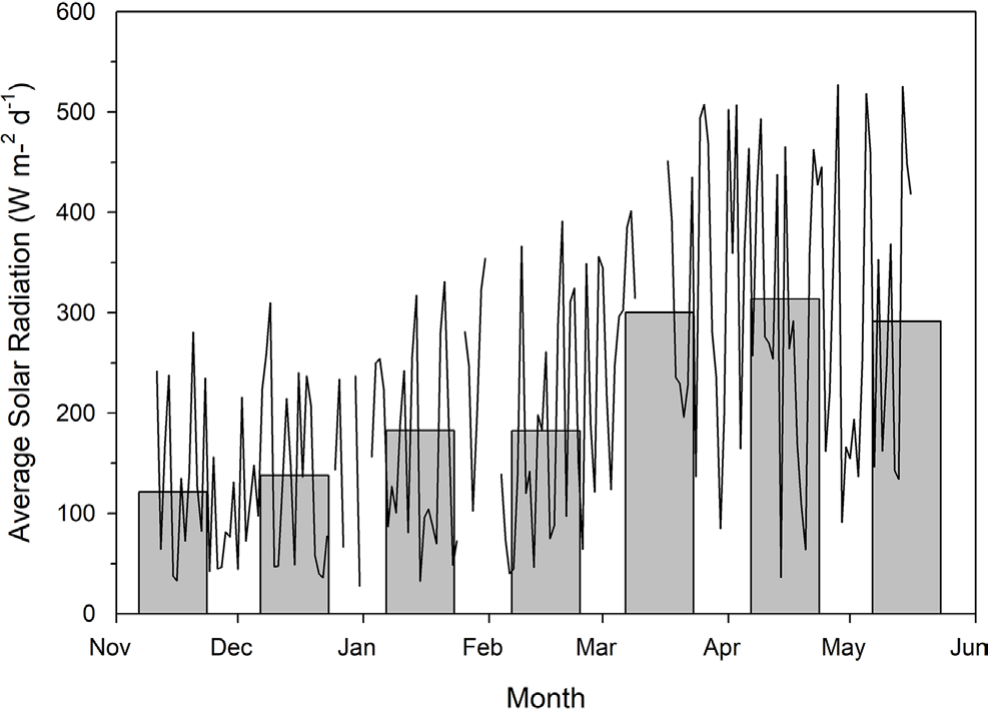
Total natural solar radiation as measured outside of the greenhouse using a Li-COR LI-200R pyranometer. Measurements were taken every 15 min between the wavelengths of 400–1100 nm throughout the course of the experiment. Measurements during the photoperiod were then averaged to provide average solar radiation readings for each individual day. The line plot represents average daily solar radiation while the bar graphs indicate average daily solar radiation throughout the month. Breaks in the line plot data indicate periods of time which were not documented due to technical malfunction.

Each greenhouses was divided into two sections *via* white curtains which were impenetrable to light. Each section was further divided into two blocks (i.e. 4 blocks per treatment). Two supplemental lighting treatments were used in the experiment: a conventional 12 h (06:00h–18:00h) lighting system providing red and blue light from LED fixtures (Pro 650e, LumiGrow Inc., Emeryville, California, USA) at the same time and a CL system with 12 h of red light during the day (06:00h–18:00h) and 12 h of blue light during the night (18:00h–06:00h) from LEDs (LumiGrow Pro 650e, **Table 1**). Low intensity of light was used during the night to prevent light pollution. Because low intensity of blue light can induce the opening of stomata (allowing photosynthesis) while high intensity of red light is needed for the opening of stomata (Shimazaki et al., 2007) and 100% blue light may increase plant height, leaf growth, and light capture (Hernández and Kubota, 2016), blue instead of red light was used during the night. The two lighting treatments provided the same DLI (**Table 1**). Application of both supplemental lighting treatments began on November 15, 2018 and continued to May 16, 2018 with harvest beginning on January 28, 2019. Throughout the experiment, supplemental lighting remained on regardless of ambient DLI (**Figure 1**) to ensure both treatments received the same total DLI. The daytime temperature was held between 21°C and 24°C depending on the ambient solar radiation while night temperature was maintained at 20 ± 0.5°C. Relative humidity of 70 ± 10% was maintained during both day and night periods. Greenhouses were CO_2_ enriched to approximately 800 µl L^−1^ when not vented.

**Table 1:** Photosynthetic photon flux density of supplemental lighting treatments during the day and night periods as measured above plant canopy (1 m below the LED fixtures).

Light treatment	06:00–18:00h		18:00h–06:00h		Daily light Integral (mol m^−2^ d^−1^)
	Red (µmol m^−2^ s^−1^)	Blue (µmol m^−2^ s^−1^)	Red (µmol m^−2^ s^−1^)	Blue (µmol m^−2^ s^−1^)	
12 h lighting	200	50	0	0	10.8
Continuous lighting	200	0	0	50	10.8

Peak outputs measured by a spectrometer (Flame spectrometer, Ocean Optics, Dunedin, FL, USA) from the red and blue LEDs were 660 nm and 447 nm, respectively.

### Growth and Destructive Measurements

Growth measurements were performed 18 days into the treatment (DIT) and included plant height, stem diameter, leaf number, leaf length, leaf diameter, and chlorophyll content of the 5^th^ leaf from 12 plants per treatment. At 55 DIT, growth measurements were again performed and included plant height, leaf number, cluster number, leaf length, width, and chlorophyll content of the 5^th^ and 10^th^ leaf from 12 plants in each treatment.

Destructive measurements were performed on eight plants per treatment 19 DIT and four plants per treatment 50 DIT. The leaf area was measured with a leaf area meter (Li-COR 3100, Li-COR Inc. Lincoln, NE, USA). The leaves, stems, and fruits, when applicable, were weighed (fresh weight) then placed in an oven at 65°C until each component was dry then weighed (dry weight).

Leaf chlorophyll was measured using a SPAD meter (model 502, Konica Minota, Osaka, Japan) and values were converted to chlorophyll content using treatment specific correction equations generated by spectrophotometric pigment analysis. Chlorophyll correction curves were generated by extracting leaf punches in 95% ethanol at 78°C for approximately 3 h until the tissue was cleared. Samples were then analyzed at 664.2, 648.6, and 470 nm wavelengths using a spectrophotometer. Concentrations of chlorophyll *a*, *b*, and carotenoids were determined *via* equations from Lichtenthaler (1978).

### Leaf Gas Exchange: Day and Night Measurements

The 5^th^ leaf was placed in the chamber of a Li-COR 6400 (Li-COR Inc. Lincoln, NE, USA) which was fitted with a 2 cm × 3 cm clear top chamber. The leaf temperature was set to 24°C with a relative humidity of 55–65% and a CO_2_ level held at 800 µl L^−1^, similar to the growth conditions. Four leaves from separate plants under each treatment were used at 20 DIT and 53 DIT for both day and night measurements. Measurements taken during the day were preformed on cloudy days to maximize the effect of supplemental lighting while minimizing the effect of natural light. Leaves were kept in the chamber until a steady-state photosynthesis rates were obtained then the average from a 2-minute period was taken.

### Leaf Gas Exchange: Light Response Curves

The 5^th^ leaf was placed in the chamber of a Li-COR 6400 which was fitted with a red/blue (88%R/12%B) LED Li-COR standard light source (2 cm × 3 cm). The leaf temperature was set to 24°C with a relative humidity of 55–65% and a CO_2_ level held at 800 µl L^−1^, similar to growth conditions. Eight leaves from separate plants under each treatment were used at 18 DIT and four leaves were used at 43 DIT. Measurements were performed on cloudy days. Light curves began at a high light intensity and decreased gradually, similar to the procedure from Evans and Santiago (2014). The light level was set to 1,500 µmol m^−2^ s^−1^ until steady-state then the light curve began with light intensity steps of: 1,500, 1,500, 1,000, 750, 500, 250, 100, 75, 50, 25, and 0 µmol m^−2^ s^−^1. At each light level, the photosynthetic rate reached a steady-state (2–4 min) then a measurement was taken for that light level. Photosynthetic rates were plotted against light intensity and fitted to a regression line following the equation y = y_o_ + a(1−e^(−b*x)^) using SigmaPlot 10.0 to determine the photosynthetic maximum. A linear regression (y = mx + b) using the photosynthetic rates between the light levels of 0–100 µmol m^−2^ s^−1^ was used to calculate both light compensation point (LCP) and quantum yield (QY).

### Leaf Gas Exchange: Co_2_ Response Curves

The 5^th^ leaf was placed in the chamber of a Li-COR 6400 which was fitted with a red/blue (88%R/12%B) LED Li-COR standard light source (2 cm × 3 cm). The leaf temperature was set to 24°C with a relative humidity of 55–65% and a light level of 250 µmol m^−2^ s^−1^. Four leaves from separate plants under each treatment were used at 18 and 43 DIT. Measurements were performed on cloudy days. Carbon dioxide response curves began at the ambient growth CO_2_ concentration (800 µl L^−1^) and reduced gradually to 0 µl L^−1^. After the 0 µl L^−1^ measurement, the CO_2_ concentration was brought back to 800 µl L^−1^ and was held steady until plant photosynthetic parameters returned to levels established during the beginning of the experiment. The CO_2_ level was then increased incrementally to 2,000 µl L^−1^ at which point the CO_2_ response curve was terminated. At each CO_2_ concentration, the photosynthetic rate reached a steady-state then a measurement was taken to produce values for that CO_2_ concentration. Photosynthetic rates were plotted against internal CO_2_ concentration (C_i_) and fitted to the FvCB model (Farquhar et al., 1980) and temperature corrected (McMurtrie and Wang, 1993; Bernacchi et al., 2001) to determine the maximum rate of photosynthesis under Rubisco-limited and RuBP-limited conditions.

### Chlorophyll Fluorescence Imaging

Intact leaflets were dark adapted using aluminium foil for 10 min. After the dark adaptation period, leaflets were detached and immediately used for chlorophyll imaging using a closed FluorCam model FC 800-C with FluorCam v.7.0 software (FluorCam, Photon System Instruments, Brno, Czech Republic). The minimum fluorescence in a dark-adapted state (F_o_) was acquired during a dark-period of 5 s, after which an 800 ms saturating light pulse (2,400 µmol m^−2^ s^−1^) from a blue LED (peak emission of 449 nm) was used to measure maximum fluorescence in a dark-adapted state (F_m_). From F_o_ and F_m_, the variable fluorescence in a dark-adapted state (F_v_) was calculated (F_v =_ F_m_ − F_o_) which was used to determine the maximum photosystem II (PSII) quantum yield (F_v_/F_m_). In general, the lower the value of F_v_/F_m_, the more severe the photoinhibition is (Baker, 2008). By calculating F_v_/F_m_ using chlorophyll fluorescence imaging, we are able to assess not only the prevalence of injury, but also the spatial heterogeneity of F_v_/F_m_ from a leaflet. Eight leaflets from the 5^th^ leaf were used for each light treatment when plants were 22 DIT. Measurements from each lighting treatment were taken again at 50 DIT with eight leaflets from the 5^th^ and 10^th^ leaves.

### Carbohydrate Analysis

Eight 0.79 cm^2^ leaf punches were taken from the 5^th^ leaf of each lighting treatment at five different time periods. Leaf punches were taken from the most distal part of the leaf at the first time point and moved towards the base of the leaf during each time point avoiding main veins. The time points were pre-night (17:45; 54 DIT), during the night (22:00; 54 DIT), pre-day (05:45; 55 DIT), mid-day (12:00; 55 DIT), and again pre-night (17:45; 55 DIT). Each punch was immediately weighed then frozen using liquid nitrogen and kept at −80°C until analysis.

Leaf punches were extracted three times in 80% boiling ethanol until tissue was cleared (Tetlow and Farrar, 1993; Lanoue et al., 2018). The ethanol soluble fraction was then dried and suspended in water and 99% chloroform (2:1 v/v), agitated, and centrifuged at 11,000 rpm to separate the water-soluble fraction (sugars) from chloroform soluble leaf components (chlorophyll, lipids, etc.). Soluble sugars were assayed using a Sucrose/Fructose/Glucose kit (Megazyme; https://www.megazyme.com) and analyzed using spectrophotometry at 340 nm.

Ethanol insoluble fractions were dried then ground and suspended in sodium acetate. Fifty microliters (∼150 U) of α-amylase was added and samples were vortexed then placed in a boiling water bath. Samples were vortexed every 4 min for 12 min. Samples were then placed in a 50°C water bath and allowed to equilibrate. Fifty micro liters (∼165 U) of amyloglucosidase was added to each sample and incubated at 50°C overnight. Thirty micro liters of each sample were then assayed in duplicate using a Total Starch Assay kit (Megazyme; https://www.megazyme.com) and analyzed using spectrophotometry at 510 nm.

### Statistical Analysis

All statistics were performed using SAS Studio 3.5. Means comparisons between 12 h lighting and CL treatments were done using a one-way ANOVA with a p < 0.05 indicating a significant difference.

## Results

### Effect of CL on Morphology and Pigments

At 18 DIT, plants exposed to CL were on average taller and had one more leaf than tomato plants under 12 h lighting (**Table 2**). At 55 DIT, plants under CL were again observed to be taller than plants under 12 h lighting indicating that CL did not hinder the plants ability to grow at a normal rate (**Table 2**). At 55 DIT, plants under 12 h lighting and CL produced similar 5^th^ leaf length and width as well as 10^th^ leaf width. The length of the 10^th^ leaf was observed to be higher in plants under 12 h lighting than plants under CL (**Table 2**). Furthermore, in contrast to measurements at 18 DIT, both plants exposed to 12 h lighting and CL produced the same number of leaves. Of note, flowers first appeared 14 DIT with no difference in flower appearance between treatments.

**Table 2:** Morphological parameters of plants grown under 12 h lighting (200 µmol m^–2^ s^–1^ red + 50 µmol m^–2^ s^–1^ blue, 06:00–18:00) and CL (200 µmol m^–2^ s^–1^ red, 06:00–18:00 + 50 µmol m^–2^ s^–1^ blue, 18:00–06:00).

Time of Measurement	18 DIT		55 DIT	
Light Treatment	12 h Lighting	Continuous lighting	12 h Lighting	Continuous Lighting
Plant Height (cm)	68.3 ± 1.6 B	79.7 ± 0.9 A	217.8 ± 2.9 B	230.4 ± 3.0 A
Stem Diameter (mm)	9.4 ± 0.1 A	9.5 ± 0.3 A	–	–
Number of Leaves/Plant	10 B	11 A	24 A	24 A
Length of 5^th^ Leaf (cm)	43.8 ± 1.0 A	41.4 ± 0.8 A	43.1 ± 0.5 A	41.1 ± 0.1 A
Width of 5^th^ Leaf (cm)	43.0 ± 2.1 A	41.3 ± 0.4 A	42.9 ± 1.0 A	43.4 ± 0.7 A
Length of 10^th^ Leaf (cm)	–	–	51.3 ± 0.4 A	48.1 ± 0.2 B
Width of 10^th^ Leaf (cm)	–	–	65.3 ± 1.4 A	61.3 ± 1.6 A

Parameters represent measurements taken at two time points during the life cycle of the plants. Values represent the mean ± the standard error of the mean with n = 4. Different letters (A, B) represent a significant difference within a time point of a given parameter at p < 0.05.

Upon destructive analysis, plants grown under supplemental CL produced more leaf area and a higher stem fresh weight at 19 DIT compared to plants grown under 12 h supplemental lighting (**Table 3**). Other destructive metrics such as leaf fresh weight, leaf dry weight, specific leaf mass, and stem dry weight were similar between the two supplemental lighting treatments (**Table 3**). At 50 DIT, plants grown under CL and 12 h lighting were similar with respect to most parameters measured during destructive analysis (**Table 3**).

**Table 3:** Destructive measurements of plants grown under 12 h lighting (200 µmol m^–2^ s^–1^ red + 50 µmol m^–2^ s^–1^ blue, 06:00–18:00) and CL (200 µmol m^–2^ s^–1^ red, 06:00–18:00 + 50 µmol m^–2^ s^–1^ blue, 18:00–06:00).

Time of measurement	19 DIT		50 DIT	
Light treatment	12 h lighting	Continuous lighting	12h lighting	Continuous lighting
Plant leaf area (cm^2^ plant^−1^)	2,860 ± 143 B	3,211 ± 175 A	12,427 ± 580 A	12,851 ± 1,004 A
Leaf fresh weight (g plant^−1^)	76.46 ± 4.57 A	82.05 ± 8.05 A	484 ± 35 A	470 ± 59 A
Leaf dry weight (g plant^−1^)	9.02 ± 0.52 A	10.12 ± 1.42 A	47.90 ± 3.02 A	46.35 ± 5.83 A
Specific leaf mass (g m^−2^)	31.43 ± 0.34 A	30.70 ± 2.77 A	38.45 ± 0.76 A	35.71 ± 2.12 A
Stem fresh weight (g plant^−1^)	116 ± 5 B	128 ± 4 A	620 ± 29	649 ± 33
Stem dry weight (g plant^−1^)	6.75 ± 0.46 A	7.85 ± 0.73 A	40.55 ± 2.57 A	41.03 ± 2.55 A
Total plant fresh biomass (g plant^−1^)	193 ± 6 A	211 ± 8 A	1104 ± 63 A	1118 ± 90 A
Total plant dry biomass (g plant^−1^)	15.75 ± 0.63 A	17.85 ± 1.40 A	88.45 ± 4.92 A	87.38 ± 8.36 A

Parameters represent measurements taken at two time points during the life cycle of the plants. Total plant fresh and dry biomass include leaves and stem weights. Any fruits presented at the 50 DIT were not included in analysis. Values represent the mean ± the standard error of the mean with n = 4. Different letters (A, B) represent a significant difference within a time point of a given parameter at p < 0.05.

At 18 DIT plants under both supplemental lighting treatments had similar concentrations of chlorophyll indicating that supplemental CL did not hinder the plants ability to produce chlorophyll or absorb light (**Table 4**). At 55 DIT the chlorophyll index of the 5^th^ leaf was similar between both supplemental lighting treatments (**Table 4**). However, the 10^th^ leaf at 55 DIT had higher values of all chlorophyll parameters measured when plants were grown under 12 h lighting compared to CL (**Table 4**). Of note, leaves from plants growing under the 12 h lighting treatment were observed to have a cupping morphology not seen in the CL treatment (**Supplementary Figure 1**).

**Table 4:** Pigment analysis of plants grown under 12 h lighting (200 µmol m^–2^ s^–1^ red + 50 µmol m^–2^ s^–1^ blue, 06:00–18:00) and CL (200 µmol m^–2^ s^–1^ red, 06:00–18:00 + 50 µmol m^–2^ s^–1^ blue, 18:00–06:00).

Time of measurement	18 DIT		55 DIT			
Light treatment	12 h Lighting	Continuous lighting	12 h Lighting		Continuous lighting	
Leaf rank	5^th^	5^th^	5^th^	10^th^	5^th^	10^th^
Chlorophyll *a* (µg cm^−2^)	49.79 ± 0.78 A	49.09 ± 0.71 A	44.34 ± 2.58 A	50.33 ± 2.03 A	43.44 ± 0.68 A	46.05 ± 0.70 B
Chlorophyll *b* (µg cm^−2^)	14.41 ± 0.15 A	14.47 ± 0.17 A	13.36 ± 0.49 A	14.53 ± 0.41 A	13.17 ± 0.15 A	13.75 ± 0.16 B
Chlorophyll *a*+*b* (µg cm^−2^)	64.21 ± 0.93 A	63.56 ± 0.88 A	57.71 ± 3.01 A	64.86 ± 2.44 A	56.60 ± 0.83 A	59.80 ± 0.86 B
Chlorophyll *a*:*b*	3.45 ± 0.02 A	3.39 ± 0.01 A	3.30 ± 0.08 A	3.46 ± 0.04 A	3.29 ± 0.01 A	3.35 ± 0.01 B
Carotenoids (µg cm^−2^)	12.16 ± 0.29 A	11.74 ± 0.19 A	10.24 ± 0.94 A	12.31 ± 0.66 A	10.18 ± 0.19 A	10.91 ± 0.19 B

Parameters represent measurements taken at two time points during the life cycle of the plants. Values represent the mean ± the standard error of the mean with n = 4. Different letters (A, B) represent a significant difference within a time point and leaf rank of a given parameter at p < 0.05.

### Physiological Responses of Tomato to CL

**Figure 2:**
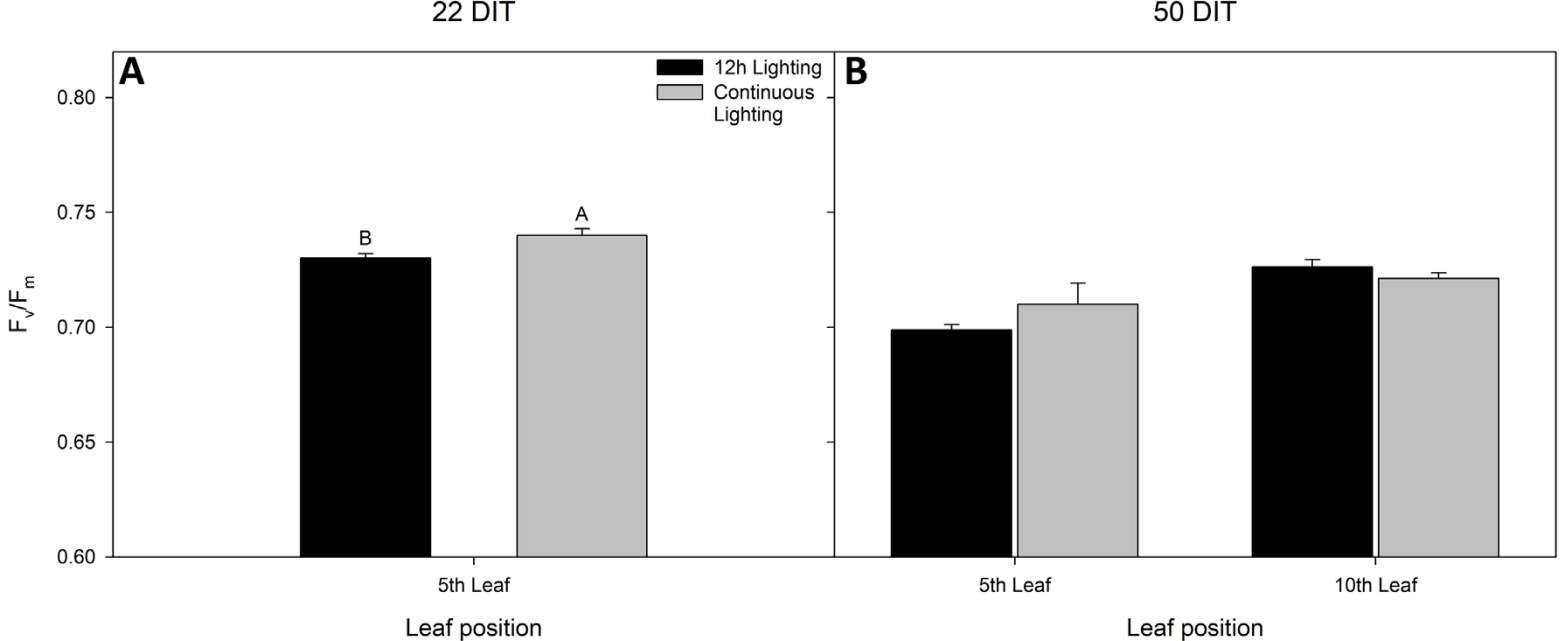
Maximum efficiency of PSII (F_v_/F_m_) from tomato leaves grown under either 12 h lighting (200 µmol m^−2^ s^−1^ red + 50 µmol m^−2^ s^−1^ blue, 06:00–18:00) and CL (200 µmol m^−2^ s^−1^ red, 06:00–18:00 + 50 µmol m^−2^ s^−1^ blue, 18:00–06:00) at 22 DIT (panel **A**) and 50 DIT (panel **B**). Of note, measurements taken at 50 DIT (panel **B**) were taken on both 5^th^ and 10^th^ leaf. Error bars represent the standard error of the mean of n = 4. Letter groups (A, B) represent significant difference between the lighting treatments at a specific time point and leaf position at p < 0.05.

**Figure 3:**
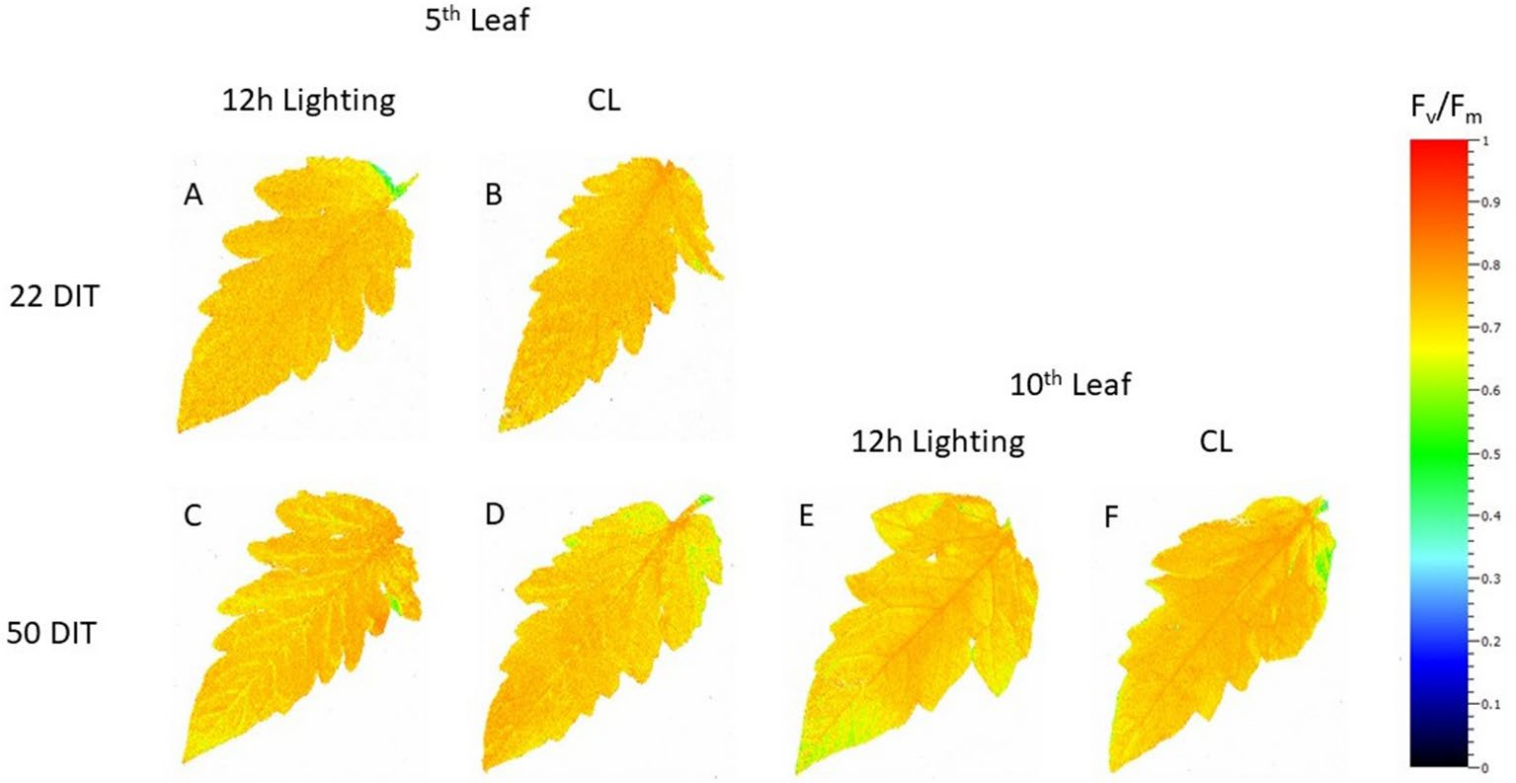
Spatial response of F_v_/F_m_ from tomato leaves grown under either 12 h lighting (200 µmol m^−2^ s^−1^ red + 50 µmol m^−2^ s^−1^ blue, 06:00–18:00) and CL (200 µmol m^−2^ s^−1^ red, 06:00–18:00 + 50 µmol m^−2^ s^−1^ blue, 18:00–06:00) at 22 DIT (A and B) and 50 DIT (C–F). Chlorophyll fluorescence images of the 5^th^ leaf are labelled (A–D) and images of the 10^th^ leaf are labelled (E and F).

At 22 DIT, the 5^th^ leaf of plants exposed to CL produced higher F_v_/F_m_ values than did leaves from 12 h lighting (**Figure 2A**). This result indicates that leaves under supplemental CL were as healthy as leaves under 12 h supplemental lighting after more than three weeks of exposure to CL. Furthermore, F_v_/F_m_ was assessed at 50 DIT on both 5^th^ and 10^th^ leaves from both supplemental light treatments. Again, leaves under CL were determined to be as healthy as leaves grown under 12 h lighting (**Figure 2B**). Of note, using chlorophyll imagining, no obvious spatial difference pertaining to F_v_/F_m_ were observed between the two lighting treatments (**Figure 3**). Under both lighting treatments, F_v_/F_m_ values in the 5^th^ leaf decreased from analysis at 22 DIT to 50 DIT (p < 0.05).

**Figure 4:**
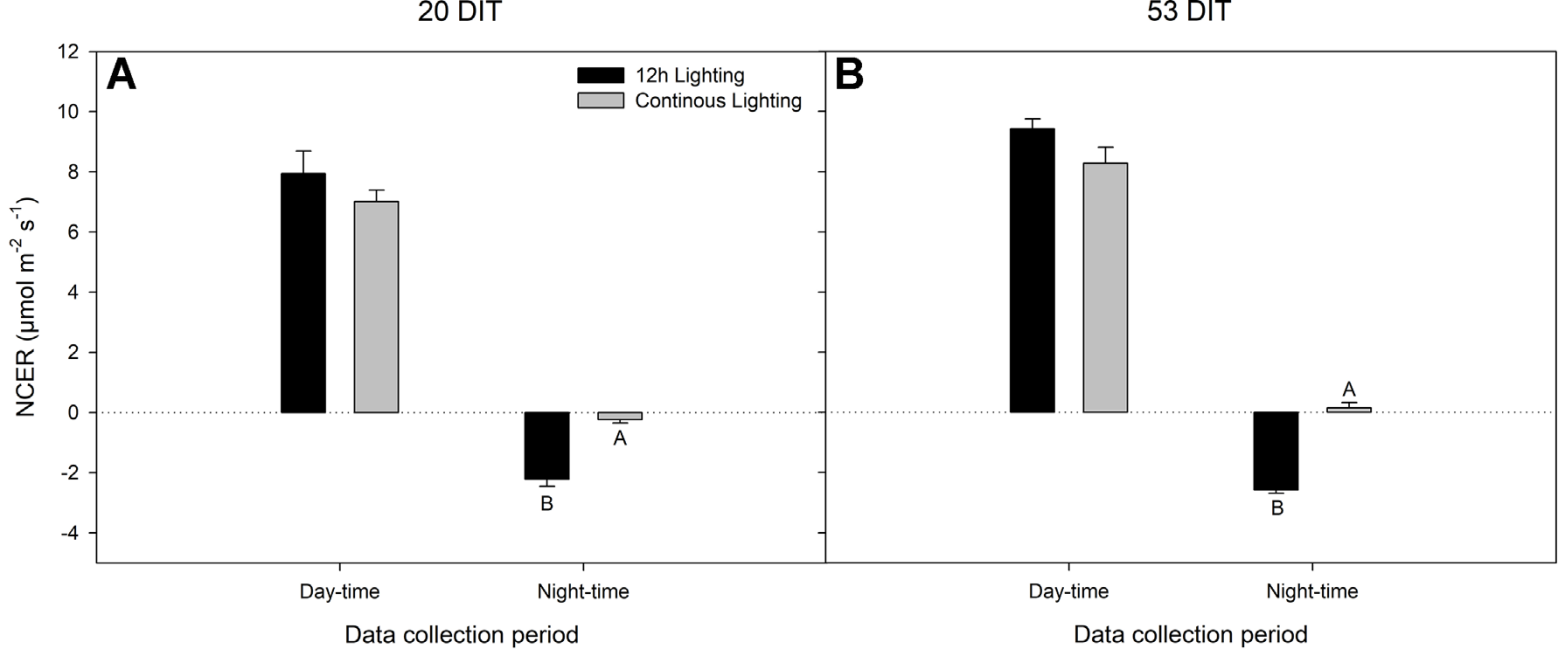
Net carbon exchange rate (NCER) of the 5^th^ leaf from tomato plants grown under 12 h lighting (200 µmol m^−2^ s^−1^ red + 50 µmol m^−2^ s^−1^ blue, 06:00–18:00) and CL (200 µmol m^−2^ s^−1^ red, 06:00–18:00 + 50 µmol m^−2^ s^−1^ blue, 18:00–06:00) at 20 DIT (panel **A**) and 53 DIT (panel **B**) during the day-time and night-time. Measurements were performed using a Li-COR 6400 fitted with a clear top chamber on a cloudy day or night and thus represent the NCER driven by the supplemental lighting. Error bars represent the standard error of the mean of n = 4. Letter groups (A, B) represent significant difference between the lighting treatments at a specific data collection period at p < 0.05.

Day-time net carbon exchange rate (NCER) at both 20 DIT and 53 DIT from leaves grown under 12 h lighting or CL produced similar values (**Figures 4A, B**). However, during the night-time (18:00h–06:00h) measurements, leaves under CL produced a higher NCER at both 20 DIT and 53 DIT (**Figures 4A, B**). Of note, during analysis at the 53 DIT period, NCER of leaves under CL during the night-time was a positive value indicating photosynthesis and a net gain in carbon instead of net loss which was seen during 12 h lighting due to respiration (**Figure 4B**). Using the NCER averages in **Figure 4**, a prediction of the total carbon gain during a 24 h period can be made. Of note, these calculations are assuming little variation in NCER over each 12 h day and night period. Nonetheless, using the day-time and night-time averages, leaves at 20 DIT and 53 DIT assimilate approximately 550 mg C m^−2^ and 800 mg C m^−2^ more respectively over a 24 h period.

**Figure 5:**
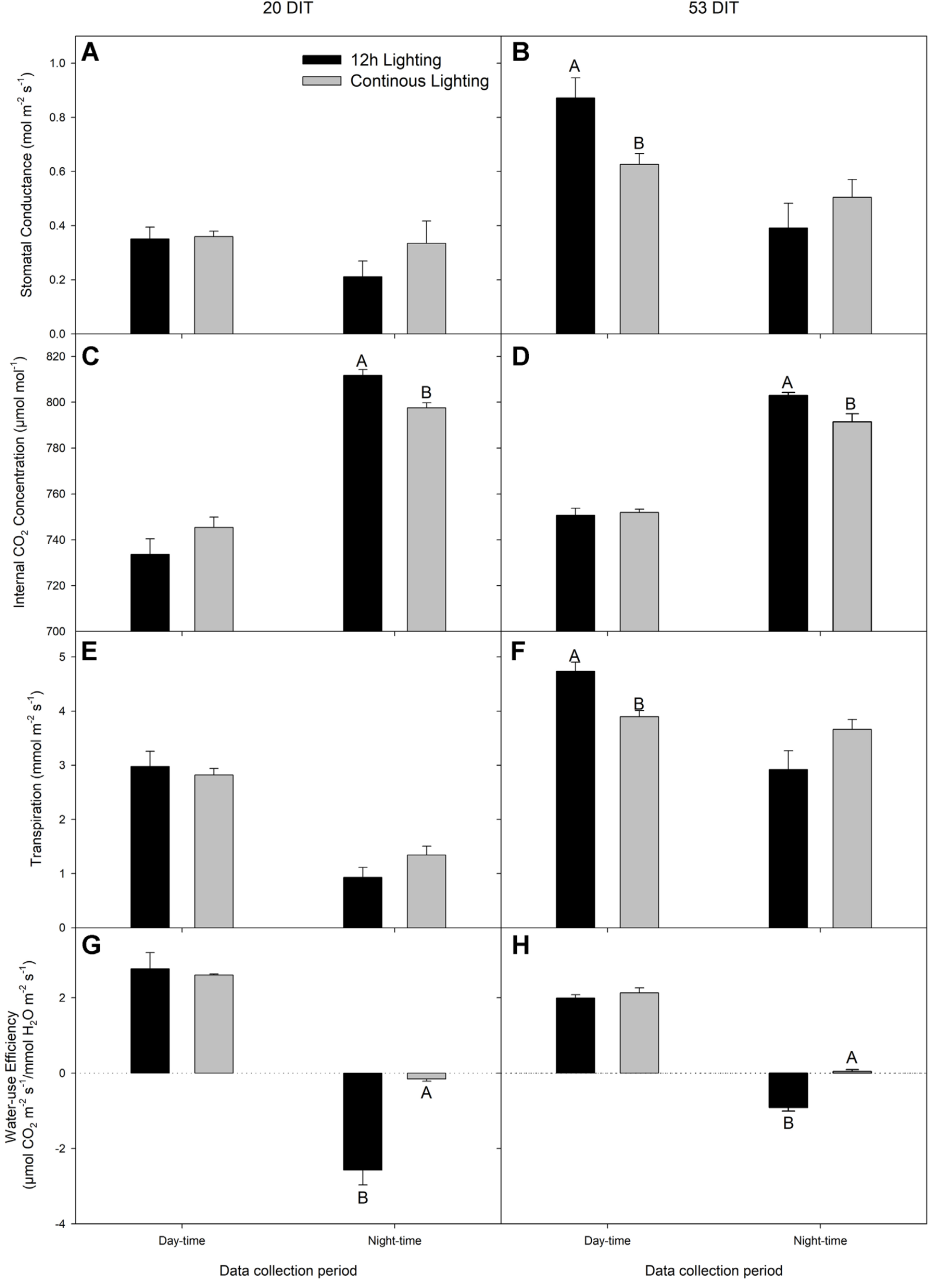
Stomatal conductance (panels **A** and **B**), C_i_ (panels **C** and **D**), transpiration (panels **E** and **F**), and water-use efficiency (panels **G** and **H**) of the 5^th^ leaf from tomato plants grown under 12 h lighting (200 µmol m^−2^ s^−1^ red + 50 µmol m^−2^ s^−1^ blue, 06:00–18:00) and CL (200 µmol m^−2^ s^−1^ red, 06:00–18:00 + 50 µmol m^−2^ s^−1^ blue, 18:00–06:00) at 20 DIT (panels **A**, **C**, **E**, and **G**) and 53 DIT (panels **B**, **D**, **E**, and **H**) during the day-time and night-time. Measurements were performed using a Li-COR 6400 fitted with a clear top chamber on a cloudy day or night and thus represent values driven by the supplemental lighting. Error bars represent the standard error of the mean of n = 4. Letter groups (A, B) represent significant difference between the lighting treatments at a specific data collection period at p < 0.05.

During day-time NCER measurements at 20 DIT, stomatal conductance, C_i_, transpiration rate, and water-use efficiency were similar between treatments (**Figures 5A, C, E, G**). During the night-time measurements, stomatal conductance and transpiration were similar between the lighting treatments while C_i_ was lower in CL leaves (**Figure 5C**) which is reflective of a higher night-time NCER (**Figure 4A**).

At 53 DIT, day-time stomatal conductance and transpiration rate were higher in leaves grown under 12 h supplemental lighting than leaves grown under supplemental CL (**Figures 5B, F**). Both C_i_ and water-use efficiency during the day-time of leaves at 53 DIT were similar between both treatments (**Figures 5D, H**). Similar to leaves at 20 DIT, leaves at 53 DIT grown under CL had a lower night-time C_i_ (**Figure 5D**) reflective of a higher night-time NCER (**Figure 4B**). Night-time stomatal conductance and transpiration rates were similar between the two lighting treatments when leaves were at 53 DIT (**Figures 5B, F**).

**Figure 6:**
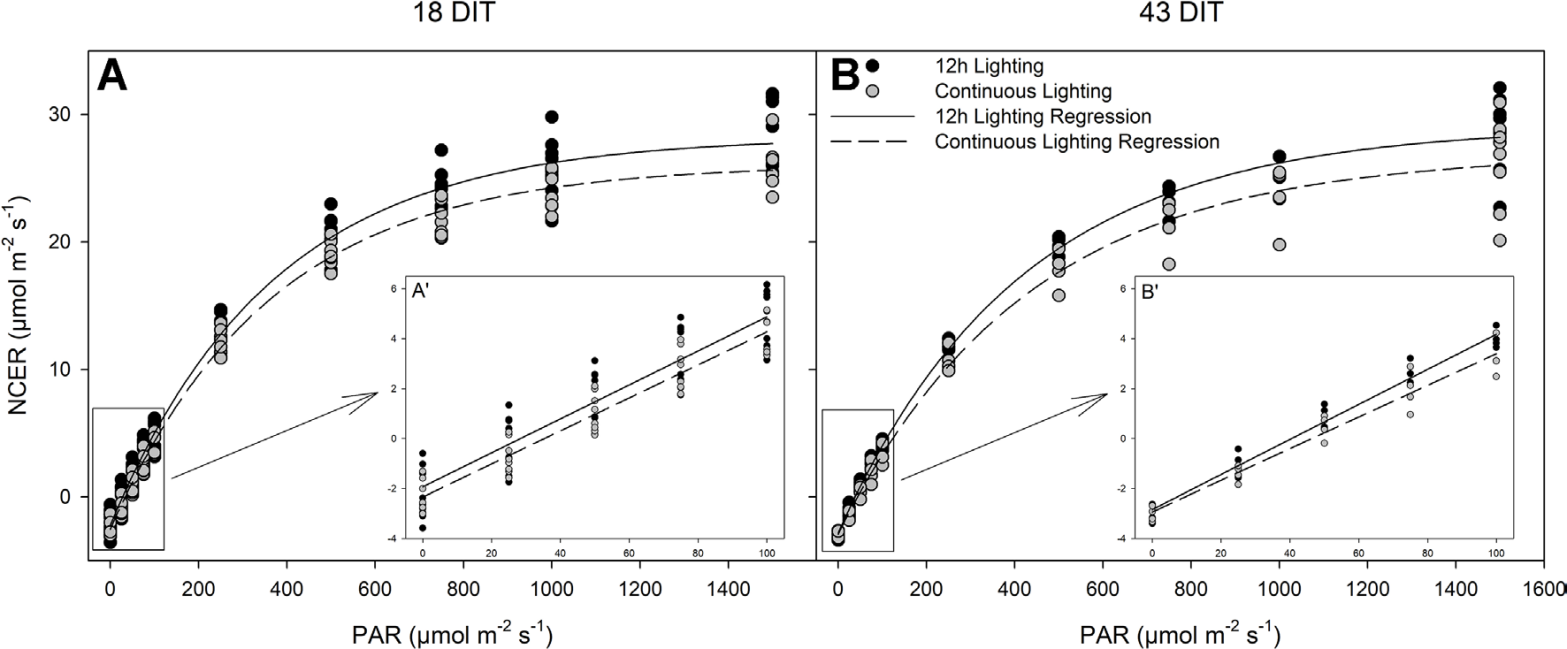
Photosynthetic light response curves from leaves grown under either 12 h lighting (200 µmol m^−2^ s^−1^ red + 50 µmol m^−2^ s^−1^ blue, 06:00–18:00) and CL (200 µmol m^−2^ s^−1^ red, 06:00–18:00 + 50 µmol m^−2^ s^−1^ blue, 18:00–06:00) at 18 DIT (panel **A)** and 43 DIT (panel **B)** as determined using a Li-COR 6400 with a red/blue standard Li-COR light source. Measurements were made at a CO_2_ concentration of 800 µl L^−1^, leaf temperature of 24°C, and a relative humidity of 55–65%. Regression lines were fit to y = y_o_ + a(1 − e^(−b*x)^) for each light treatment. Insert A’ and B’ are magnifications of 0–100 µmol m^−2^ s^−1^ PAR regions fit to the regression line y = mx + b.

All parameters, both measured (respiration) and calculated [light compensation point, quantum yield, and photosynthetic maximum (Pn_max_)] were similar between lighting treatments at both time points studied (**Table 5** and **Figure 6**). Furthermore, all relevant photosynthetic parameters (day-time NCER, quantum yield, and Pn_max_) were quantified on a chlorophyll basis, again producing similar values between the two lighting treatments (P < 0.05; data not shown).

**Table 5:** Summary of the major physiological traits as determined by leaf light response curves (**Figure 6**) from tomatoes grown under 12 h lighting (200 µmol m^–2^ s^–1^ red + 50 µmol m^–2^ s^–1^ blue, 06:00–18:00) and CL (200 µmol m^–2^ s^–1^ red, 06:00–18:00 + 50 µmol m^–2^ s^–1^ blue, 18:00–06:00).

Time of measurement	Light treatment	Respiration (µmol CO_2_ m^−2^ s^−1^)	Light compensation point (µmol m^−2^ s^−1^)	Quantum yield (µmol CO_2_ m^−2^ s^−1^/µmol m^−2^ s^−1^)	Pn_max_ (µmol CO_2_ m^−2^ s^−1^)
18 DIT	12 h lighting	−2.03 ± 0.38 A	28.83 ± 5.98 A	0.068 ± 0.001 A	30.61 ± 1.11 A
	Continuous lighting	−2.36 ± 0.23 A	35.62 ± 3.81 A	0.066 ± 0.001 A	27.94 ± 1.21 A
43 DIT	12 h lighting	−2.96 ± 0.19 A	40.45 ± 3.11 A	0.070 ± 0.001 A	32.06 ± 1.83 A
	Continuous lighting	−2.94 ± 0.15 A	46.84 ± 3.44 A	0.063 ± 0.005 A	29.98 ± 2.24 A

Respiration values were the averages of NCER when the light level was 0 µmol m^−2^ s^−1^. The light compensation point and quantum yield were calculated from a regression line (y = mx + b) fitted to the values between the PAR values of 0–100 µmol m^−2^ s^−1^. The photosynthetic maximum (Pn_max_) was calculated from y = y_o_ + a(1 − e^(−b*x)^). Values represent the mean ± the standard error of the mean with n = 4. The same letter (A) represents a no significant difference within a time point and of a given parameter at p < 0.05.

**Figure 7:**
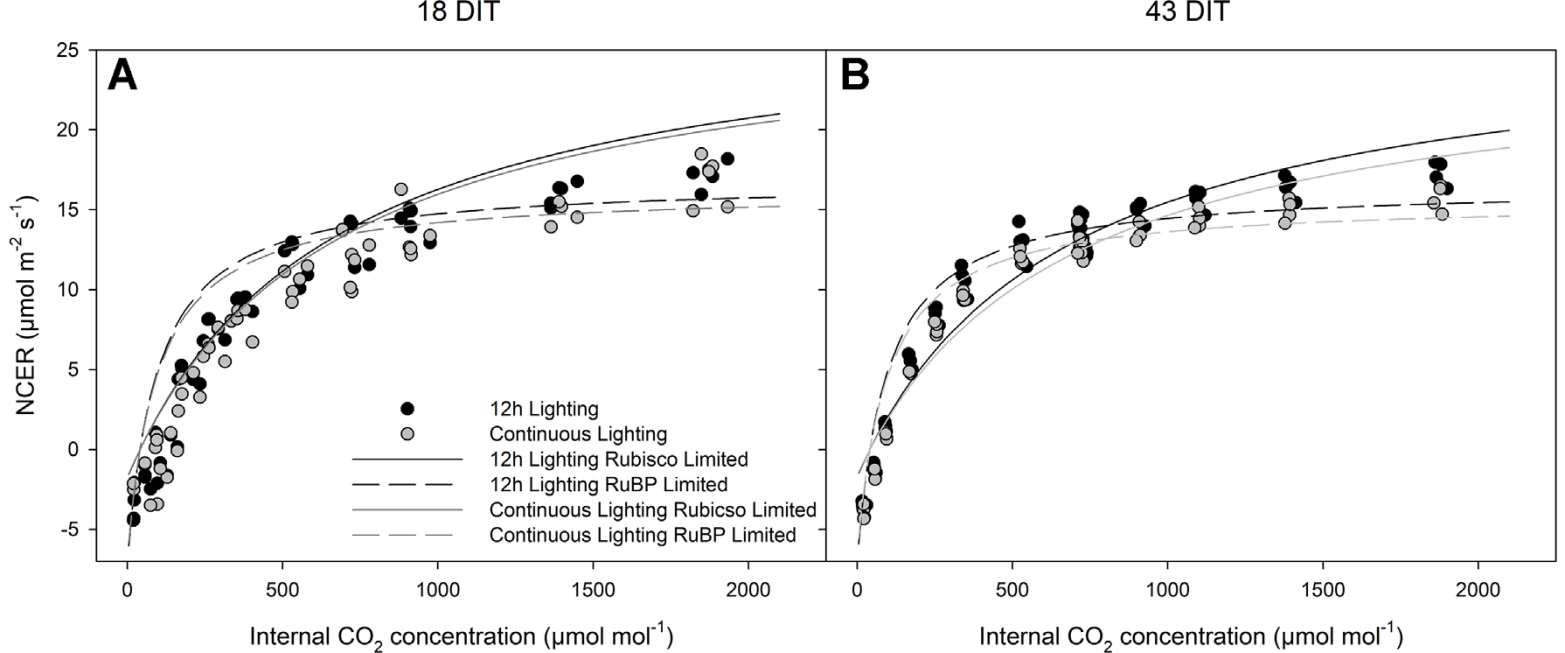
Photosynthetic CO_2_ response curve from leaves grown under either 12 h lighting (200 µmol m^−2^ s^−1^ red + 50 µmol m^−2^ s^−1^ blue, 06:00–18:00) or CL (200 µmol m^−2^ s^−1^ red, 06:00–18:00 + 50 µmol m^−2^ s^−1^ blue, 18:00–06:00) at 18 DIT (panel **A**) and 43 DIT (panel **B** as determined using a Li-COR 6400 with a red/blue standard Li-COR light source. Measurements were made at 250 µmol m^−2^ s^−1^ PAR, a temperature of 24°C, and relative humidity of 55–65%. Rubisco and RuBP limited fit lines were determined using temperature corrections from McMurtrie and Wang (1993) and Bernacchi et al. (2001).

**Figure 7** represents the CO_2_ response curve at two time points from leaves of plants grown under 12 h supplemental lighting or supplemental CL. These response curves were specifically run at a light level of 250 µmol m^−2^ s^−1^ to determine the response of leaves from both lighting treatments at or near their growth conditions (**Figure 7**). Similarities between 12 h lighting and CL treatments indicate no affect of CL on photosynthesis.

### Carbohydrate Analysis of Tomatoes Under CL

**Figure 8:**
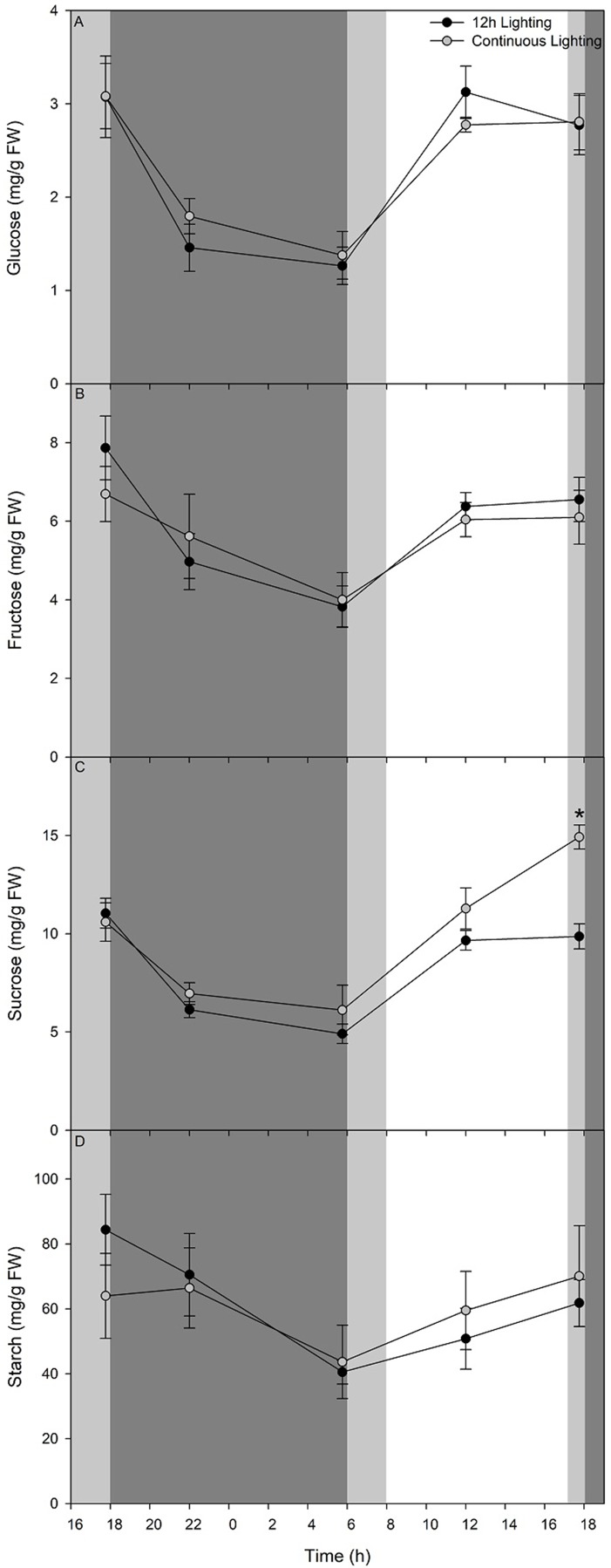
Diurnal pattern of glucose (panel **A**), fructose (panel **B**), sucrose (panel **C**), and starch (panel **D**) from the 5^th^ leaf from plants grown under either 12 h lighting (200 µmol m^−2^ s^−1^ red + 50 µmol m^−2^ s^−1^ blue, 06:00–18:00) and CL (200 µmol m^−2^ s^−1^ red, 06:00–18:00 + 50 µmol m^−2^ s^−1^ blue, 18:00–06:00) on the 54^th^ and 55^th^ DIT. The dark grey area represents the period of darkness during the 12 h lighting treatment and the period of blue light during the CL treatment. The light grey area represents the natural photoperiod. Eight 0.79 cm^2^ leaf punches were taken from the 5^th^ most fully expanded leaves during different time points over a 24 h period. Error bars represent the standard error of the mean of n = 4. An asterisk (*) represents a significant difference between the lighting treatments at a specific time point within a graph at p < 0.05.

Carbohydrate status (**Figures 8A–C**) shows a rapid decrease of glucose, fructose, and sucrose respectively during the initial 4 h during the dark period of the 12 h lighting treatment and the blue light period of the CL treatment. A decrease in these three sugars continues until 05:45 just before the lights were turned on in the 12 h lighting treatment or the blue light became red light in the CL treatment (**Figures 8A–C**). Upon illumination or a change in wavelength accompanied by an increase in light intensity, glucose, fructose, and sucrose levels accumulate during the initial 6 h to levels comparable with pre-night levels (**Figures 8A–C**). Both glucose and fructose levels remain steady from 12:00–17:45 (**Figures 8A, B**). It is important to note that while supplemental lighting treatment photoperiods were 06:00–18:00 for 12 h lighting or continuous lighting, the natural solar photoperiod during the time of carbohydrate measurements was 08:00–17:12. Patterns of glucose and fructose, sucrose levels were similar between sampling at 12:00 and 17:45 in leaves grown under 12 h lighting (**Figure 8C**). However, leaves grown under CL continued to accumulate sucrose during the final hours under red light and had higher sucrose levels at 17:45 at 55 DIT compared to leaves exposed to 12 h supplemental lighting (**Figure 8C**).

Throughout the night period of the 12 h supplemental lighting treatment, starch levels within leaves decreased during the first 4 h (**Figure 8D**). Interestingly, during the initial 4 h of the blue light period of the CL treatment, starch levels remained similar to those from leaves sampled before the light spectral switch (17:45; **Figure 8D**). From 22:00 at 54 DIT to 05:45 at 55 DIT starch levels in both 12 h lighting and CL treatments decreased (**Figure 8D**). During the subsequent light period, starch levels of leaves from both 12 h lighting and CL treatments increased at similar rates returning to levels comparable to those from leaves sampled at 17:45 at 54 DIT (**Figure 8D**).

### Fruit Yield

During the initial sampling period (January 28, 2019–February 21, 2019) 75–99 DIT, fruit yield (total fruit fresh weight) was higher when plants were grown under CL (**Table 6**). During this same sampling period, fruit number and average fruit weight (size) were similar between the treatments. During the remainder of the sampling periods (February 22, 2019–May 16, 2019) fruit yield, number, and average fruit weight (size) did not differ between the two treatments within a sampling period (**Table 6**).

**Table 6:** Tomato fruit yield from plants grown under 12h supplemental lighting or supplemental continuous lighting from January 28, 2019 to May 16, 2019.

Light treatment	Sampling period (4 weeks)	Fruit fresh weight (kg m^−2^)	Fruit number (# m^−2^)	Average fruit weight (g fruit^−1^)
12 h lighting	1	6.49 ± 0.13 B	56.0 ± 2.7 A	115.88 ± 3.55 A
	2	9.09 ± 0.49 A	73.7 ± 1.0 A	123.35 ± 5.15 A
	3	11.88 ± 0.24 A	84.6 ± 2.2 A	140.43 ± 5.18 A
	4	11.94 ± 0.51 A	83.3 ± 2.3 A	143.42 ± 4.20 A
Continuous lighting	1	7.49 ± 0.22 A	60.8 ± 3.0 A	123.15 ± 6.02 A
	2	8.55 ± 0.47 A	73.6 ± 1.5 A	116.16 ± 5.47 A
	3	10.93 ± 0.64 A	81.1 ± 2.4 A	134.71 ± 4.27 A
	4	11.71 ± 0.60 A	81.7 ± 1.0 A	143.68 ± 6.30 A

Sampling periods (every 4 weeks) correspond to January 28, 2019–February 21, 2019 (1), February 22, 2019–March 21, 2019 (2), March 22, 2019–April 23, 2019 (3), and April 24, 2019–May 16, 2019 (4). Within each parameter and sampling period, different letters (A, B) represent statistically significant difference at p < 0.05.

## Discussion

Plant biomass during controlled environment production is largely dictated by the total amount of the light intercepted by the plant [daily light integral (DLI) – intensity × photoperiod]. Continuous light (CL, 24 h lighting) could increase plant biomass and yield if CL does not cause any injury. It is also more economical because the costs of the light fixtures stay the same, cost of electricity in Ontario, Canada at night is lower, and heat released by the light fixtures help to meet the heating requirement during the night. In this study, we set out to determine the effects of alternating spectrum supplemental CL on greenhouse grown tomatoes. Overall, the results presented in this paper indicate that tomato plants grown under supplemental CL with alternating red and blue spectrum do not show signs of injury such as the leaf chlorosis observed in other studies investigating CL (Hillman, 1956; Matsuda et al., 2014; Haque et al., 2015; Haque et al., 2017; Velez-Ramirez et al., 2017b). Furthermore, the morphological and physiological parameters examined were similar to tomato plants grown under a conventional 12 h supplemental lighting regime.

Most studies investigated the effect of CL on tomato were preformed inside growth chambers which lack the normal light/dark cycle of natural lighting (Matsuda et al., 2014; Haque et al., 2015; Haque et al., 2017; Velez-Ramirez et al., 2017b). However, Arthur et al. (1930), Demers et al. (1998) and Hao et al. (2017a) studied tomatoes produced in greenhouses under supplemental MH or HPS lights and still observed CL-injury. This indicates that the oscillations present during a natural light/dark period do not reduce the presence of CL-injury in tomato plants.

The introduction of cost-effective LEDs has allowed for the wavelength specific modification of morphology and physiology in tomatoes (Hernández and Kubota, 2012; Liu et al., 2012; Lanoue et al., 2018). In our study, wavelength specific LEDs were utilized to create an alternating spectrum CL regime which produced injury-free tomato plants. The utilization of LEDs to produce an alternating spectrum during CL has been previously documented in lettuce (Ohtake et al., 2018). In this study using lettuce, an alternating CL spectrum with red light during the day (12 h) and blue light during the night (12 h) produced the highest dry weight and leaf area compared to a CL with a constant red/blue spectrum and a CL provided by fluorescent lights (Ohtake et al., 2018).

During the initial stages of our experiment (late November–early December), the natural light intensity was low, and the natural photoperiod is short, thus, supplemental lighting had the largest effect. Similar to results in Ohtake et al. (2018) using lettuce, during this period, plants grown under CL had a larger leaf area and taller stem height than plants grown under 12 h lighting. Since day-time NCER are similar between the two treatments, it is likely that elevated NCER between 18:00–06:00 from the CL treatment allocated more carbon for vegetative growth (**Figure 4**). As previously theorized, growing tomatoes under CL during the light limiting months in winter can lead to increased biomass accumulation when CL-injury is not present (Velez-Ramirez et al., 2012).

During a similar early stage of this experiment, total fruit production (yield) increased in plants grown under CL (**Table 6**). During the subsequent months, fruit yield was similar between the two treatments. As noted above, the first period of production was characterized with low natural light intensity and short photoperiod, allowing supplemental light to have a maximized effect. Plant grown under CL during this period had a higher initial leaf area and higher stem height (**Tables 2**, **3**). These characteristics can lead to a higher light capture which may be able to support enhanced fruit production. Furthermore, a recent study indicated that sole blue light was able to increase carbon export, the process responsible for transporting photo-assimilate from the leaf to growing sinks such as fruit (Lanoue et al., 2018). Thus, the sole blue light during the CL night period may allow for increased carbon export leading to enhanced fruit production. As the natural DLI increased throughout the experiment, this advantage from growth under CL may diminish leading to comparable fruit production later in the experiment (**Table 6**). Thus, CL may be most beneficial during periods of low natural lighting and less beneficial as solar DLI increases.

Leaf carbohydrate status plays an important role in regulating photosynthetic performance (Azcón-Bieto, 1983). Continuous lighting has been shown to down-regulate genes related to photosynthesis within tomato leaves (Velez-Ramirez et al., 2014). The down-regulation has been associated with excess starch and sucrose levels leading to oxidative stress within the leaf, causing CL-injury (Velez-Ramirez et al., 2017a). The excess accumulation is likely due to the constant energy influx due to light availability during what would be the night period during a non-CL photoperiod.

Within our CL regime, leaves produced similar concentrations, as well as diurnal patterns of glucose, fructose, sucrose, and starch as leaves grown under a 12 h photoperiod (**Figure 8**). These results may be explained by one of two factors. Potentially, plants grown under CL with alternating red and blue LEDs are able to control their carbohydrate metabolism similarly to plants under a 12 h photoperiod. Or, the low light intensity used during the subjective night period did not drive photosynthesis at high enough levels for carbohydrates to build up within the leaf. Regardless of the mechanism at work, excess starch and sucrose were not able to build up in the leaf, end product feedback inhibition of photosynthesis was alleviated and CL-injury symptoms were not present.

Furthermore, parameters related to photosynthetic performance (i.e. Pn_max_, quantum yield, and F_v_/F_m_) of leaves grown under CL were similar to leaves grown under 12 h lighting. Similar results were observed in other studies when a temperature drop was performed, which reduced or alleviated CL-injury (Haque et al., 2015; Hao et al., 2017a; Hao et al., 2017b; Haque et al., 2017; Hao et al., 2018b). Thus, alternating the light spectrum during a CL regime maintains normal leaf function, a result which has not previously been reported during CL under constant spectrum within tomatoes unless a temperature drop was present (Arthur et al., 1930; Demers et al., 1998; Matsuda et al., 2014; Haque et al., 2015; Haque et al., 2017; Velez-Ramirez et al., 2017a).

Unlike other studies (Velez-Ramirez et al., 2014; Haque et al., 2015; Matsuda et al., 2016) our study used a low blue light level during the subjective night period of the CL treatment. The light level used produced a photosynthetic level near the compensation point alleviating excess carbohydrate accumulation (**Figure 8**). Furthermore, low blue light level may initiate gene turnover similar to what would occur during the shift to a night period in a conventional light treatment (Velez-Ramirez et al., 2014). Perhaps the drastic shift from red to blue light reduces photo-oxidative stressed caused by continuous illumination with one wavelength *via* phytochrome and cryptochrome genetic regulation (Casal, 2000). However, exact mechanisms involved require further experimentation pertaining to wavelength specific genetic regulation.

The importance of understanding the role of photoreceptors in physiological responses has increased since the introduction of wavelength specific LED lighting options. A recent study suggests the role of phytochrome (PHY) A in eradicating CL-injury in tomato (Velez-Ramirez et al., 2019). Phytochromes respond to red and far-red light while cryptochromes perceive blue light. In our study, we showed that the implementation of an alternating spectrum CL using red LED light during the daytime and blue LED light during the night, allowed for injury-free tomato production. Thus, the use of low intensity of blue light during the night might not disturb circadian rhythms. Phytochromes are activated by red light and have been shown to play a key role in anthocyanin biosynthesis (PHY A at low light levels and PHY B1 at higher) in tomato (Weller et al., 2000). Anthocyanins have been shown to have a photoprotective role and help re-establish balance between light capture and CO_2_ fixation, thus reducing the potential for photo-oxidative damage (Steyn et al., 2002). Furthermore, under low irradiance blue light, similar to that used in this study, PHY A retains its function and continues to promote anthocyanin biosynthesis (Weller et al., 2000). Taken together, the role of PHY B1 during high light periods and PHY A during low light promote anthocyanin biosynthesis in tomato leading to a potential photoprotection mechanism during our CL regime alleviating injury.

Another hypothesis could be that an alternating spectrum CL regime could maintain normal circadian rhythms, something which probably did not occur when no change in spectrum was used (Velez-Ramirez et al., 2017a). The drastic shift in spectrum from red and sunlight to 100% blue may initiate gene turn over *via* photoreceptor signal transduction including important genes involved in carbohydrate status or light harvesting such as *cab13* (Velez-Ramirez et al., 2014). In this way, an alternating spectrum during CL may act similarly to a drastic temperature drop as seen in other studies (Haque et al., 2015; Haque et al., 2017). Regardless of the mechanism, the role of photoreceptors in CL-injury is interesting and requires further research.

## Conclusion

Taken together, the results indicate that using alternating red and blue (100% during the night) spectrum CL allows for injury-free tomato production. Morphologically, plants grown under CL were similar to plants grown under the 12 h lighting treatment in most aspects. All physiological parameters (Pn_max_, F_v_/F_m_, respiration rates, and stomatal conductance) were similar between leaves grown under CL and 12 h lighting. One noticeable difference was the NCER between 18:00–06:00. During this period, leaves under the CL treatments produced higher NCER values and produced positive values at 53 DIT indicating carbon gain during the subjective night period. Carbohydrate diurnal patterns were similar between both light treatments displaying a drop during between 18:00 and 06:00 and a rise between 06:00 and 18:00. This result indicates that the CL with alternating red and blue allows for a normal circadian rhythm with regards to carbohydrate metabolism. The effects of spectral quality and timing of light quality during CL is poorly understood. Our study indicates that an alternating red (200 µmol m^−2^ s^−1^) and blue (50 µmol m^−2^ s^−1^) CL regime allow for injury-free tomato production. Thus, the need for further research pertaining to spectral quality and the role of photoreceptors during CL tomato production is needed.

## Data Availability

All datasets generated for this study are included in the manuscript and the **Supplementary Files**.

## Author Contributions

XH (corresponding author) developed the concept and designed the lighting strategy for the study and revised the manuscript. JL developed data collection protocols, acquired and analyzed the physiological and growth data, and drafted and revised the manuscript. JZ managed the greenhouse trial and contributed in plant growth data collection. CL and AT contributed in plant growth and physiological data collection. BG revised the manuscript.

## Conflict of Interest Statement

The authors declare that the research was conducted in the absence of any commercial or financial relationships that could be construed as a potential conflict of interest.
