# Supplementary material for: Alternating Red and Blue Light-Emitting Diodes Allows for Injury-Free Tomato Production With Continuous Lighting
Source: Front Plant Sci. 2019 Sep 13;10:1114. doi: 10.3389/fpls.2019.01114 (PMC6754077; doi:10.3389/fpls.2019.01114)
Supplement: Supplementary file 1 [file Table_1.docx]

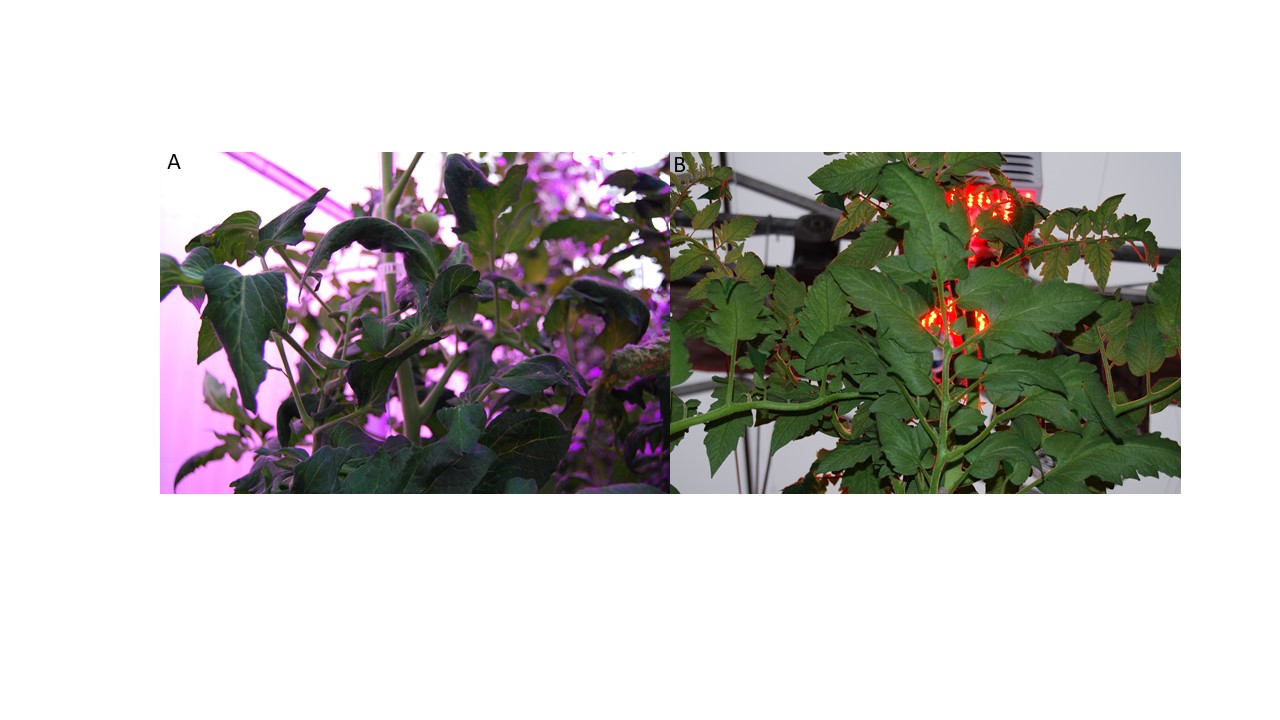


**Supplementary Figure 1:** Leaf morphology of plants under 12h supplemental lighting (panel A) and CL (panel B). The red circle highlights leaf cupping of leaflets grown under 12h supplemental red/blue LED lighting while leaves under CL remain flat.
